# Carbonated tiger-high above-ground biomass carbon stock in protected areas and corridors and its observed negative relationship with tiger population density and occupancy in the Terai Arc Landscape, Nepal

**DOI:** 10.1371/journal.pone.0280824

**Published:** 2023-01-25

**Authors:** Kanchan Thapa, Gokarna Jung Thapa, Ugan Manandhar, Maheshwar Dhakal, Shant Raj Jnawali, Tek Narayan Maraseni

**Affiliations:** 1 WWF Nepal, Baluwatar, Kathmandu, Nepal; 2 Institute for Biodiversity and Ecosystem Dynamics, University of Amsterdam, Amsterdam, The Netherlands; 3 Department of National Parks and Wildlife Conservation, Babarmahal, Kathmandu, Nepal; 4 University of Southern Queensland, Institute for Life Sciences and the Environment, Toowoomba, Queensland, Australia; Amity University, INDIA

## Abstract

Healthy natural forests maintain and/or enhances carbon stock while also providing potential habitat and an array of services to wildlife including large carnivores such as the tiger. This study is the first of its kind in assessing relationships between above-ground biomass carbon stock, tiger density and occupancy probability and its status in protected areas, corridors, and forest connectivity blocks. The dataset used to assess the relationship were: (1) Converged posterior tiger density estimates from camera trap data derived from Bayesian- Spatially Explicit Capture-Recapture model from Chitwan National Park; (2) Site wise probability of tiger occupancy estimated across the Terai Arc Landscape and (3) Habitat wise above-ground biomass carbon stock estimated across the Terai Arc Landscape. Carbon stock maps were derived based on eight habitat classes and conservation units linking satellite (Landsat 7 ETM+) images and field collected sampling data. A significant negative relationship (r = -0.20, *p*<0.01) was observed between above-ground biomass carbon stock and tiger density in Chitwan National Park and with tiger occupancy (r = -0.24, *p* = 0.023) in the landscape. Within protected areas, we found highest mean above-ground biomass carbon stock in high density mixed forest (~223 tC/ha) and low in degraded scrubland (~73.2 tC/ha). Similarly, we found: (1) highest tiger density ~ 0.06 individuals per 0.33 km^2^ in the riverine forest and lowest estimates (~0.00) in degraded scrubland; and (2) predictive tiger density of 0.0135 individuals per 0.33 km^2^ is equivalent to mean total of 43.7 tC/ha in Chitwan National Park. Comparatively, we found similar above-ground biomass carbon stock among corridors, large forest connectivity blocks (~117 tC/ha), and within in tiger bearing protected areas (~119 tC/ha). Carbon conservation through forest restoration particularly in riverine habitats (forest and grassland) and low transitional state forests (degraded scrubland) provides immense opportunities to generate win-win solutions, sequester more carbon and maintain habitat integrity for tigers and other large predators.

## Introduction

The amount of carbon stored in terrestrial vegetation is a key component of the global carbon cycle [1]. Variation in carbon flux has been well understood in the terrestrial ecosystem [2] and forests have been absorbing twice as much carbon as they emitted [3]. For example, from 2001 to 2019, global forests emitted 8.25 GtCO_₂_e yr^-1^ and removed 15.6 GtCO_₂_e yr^-1^ [4]. Globally, there is an important positive to weak relationship between carbon density and biodiversity [5–7]. Conservation of the forest ecosystem is critical for maintaining both the biodiversity richness and carbon density including sequestering the atmospheric carbon [8].

Large mammals play a key role in shaping and maintaining natural processes [9, 10]; ecoregions that retain intact large mammalian faunas also sequester large amounts of carbon [11]. Elimination of large vertebrates may adversely affect ecosystem dynamics [10] and ultimately reduce carbon sequestration and other ecosystem services [12]. Tiger (*Panthera tigris*) has been identified as one of the large mammals and top predators in the trophic structures whose density and abundance affects the ecosystem and supports ecosystem services [13]. Protection of carbon-rich ecosystems such as forest ecosystems within the tiger conservation landscapes [14], can directly or indirectly benefit overall diversity including wildlife [15, 16]. Wikramanayake et al. [17] classified carbon rich ecosystems into pioneer sal forests, mixed forests, riparian forests, alluvial floodplain, and grasslands distributed along the lowland plains (Tarai) and churia (Siwalikh, also called as chure) physiographic range as potential habitats for tigers. Terai Arc Landscape (TAL) is a pioneer transboundary tiger conservation landscape spatially distributed between Nepal and India. Managing the tiger habitat is highly pressing in the premises of doubling the wild tigers in its ranging states and Nepal is the first country in the world to double its tiger populations in wild by 2022 [18]. Thus, there is a dire need for conservation that integrates and brings in the synergies between conservation of tiger, its habitat and carbon it contains measured in-terms of above-ground biomass carbon stock (hereafter referred to as AGBCS).

It has been shown that carbon density in tiger conservation forest landscapes is 3.5 times greater than in forests occurring outside tiger landscapes [12, 19]. Gurung et al. [20] estimated a 252,000 ± 22,000 Gg C stock contain within the potential tiger habitat that hoards high tiger densities ($\hat{D}$~1–5 tigers per 100 km^2^) within the five protected areas and landscape occupancy probabilities (ψ~0.68) [21] in TAL-Nepal. There remains a knowledge gap on empirical relationship between tiger and carbon it contains within its potential habitat in the landscape and elsewhere for bringing synergies between goal of conserving tiger and securing ecosystem services. In this connection, this research focused on exploring the relationship between healthy tiger population-in terms of population densities [22] and occupancy probabilities [23]- and AGBCS in its potential tiger habitat.

We reviewed the readily available datasets to assess the relationship between Spatially Explicit Capture-Recapture (SECR) tiger density and occupancy probabilities with AGBCS. Reduction in grassland patch along with increase in forest patch in Chitwan National Park (CNP) may negatively affect the abundance of the prey species, resulting in limiting tiger population density [24]. Restoring the habitat heterogeneity is the best approach for managing habitat, benefiting the tiger and its prey [24]. We hypothesized that tiger density and occupancy probabilities (Psi, ψ, 0–1) would follow a negative relationship with AGBCS mediated by habitat classes identified along the potential tiger habitat in the landscape. In this paper, we assessed: the relationship between tiger density and occupancy probabilities and AGBCS; and AGBCS status in eight habitat types identified in the landscape and conservation units (protected areas, corridors, and large forest connectivity blocks) defined for tiger conservation.

## Materials and methods

### Ethics statement

The study was conducted based on output result data sets from National Tiger survey conducted in 2013 and above-ground carbon stock estimation survey conducted in Nepal side of TAL. Dataset used for the analysis can be reused with due permission and acknowledgement from Department of National Park and Wildlife Conservation and WWF Nepal. Animal care and use committee approval was not required as it was based on the secondary data that used non-invasive techniques such camera trapping and sign surveys.

### Study areas and sites

**Fig 1:**
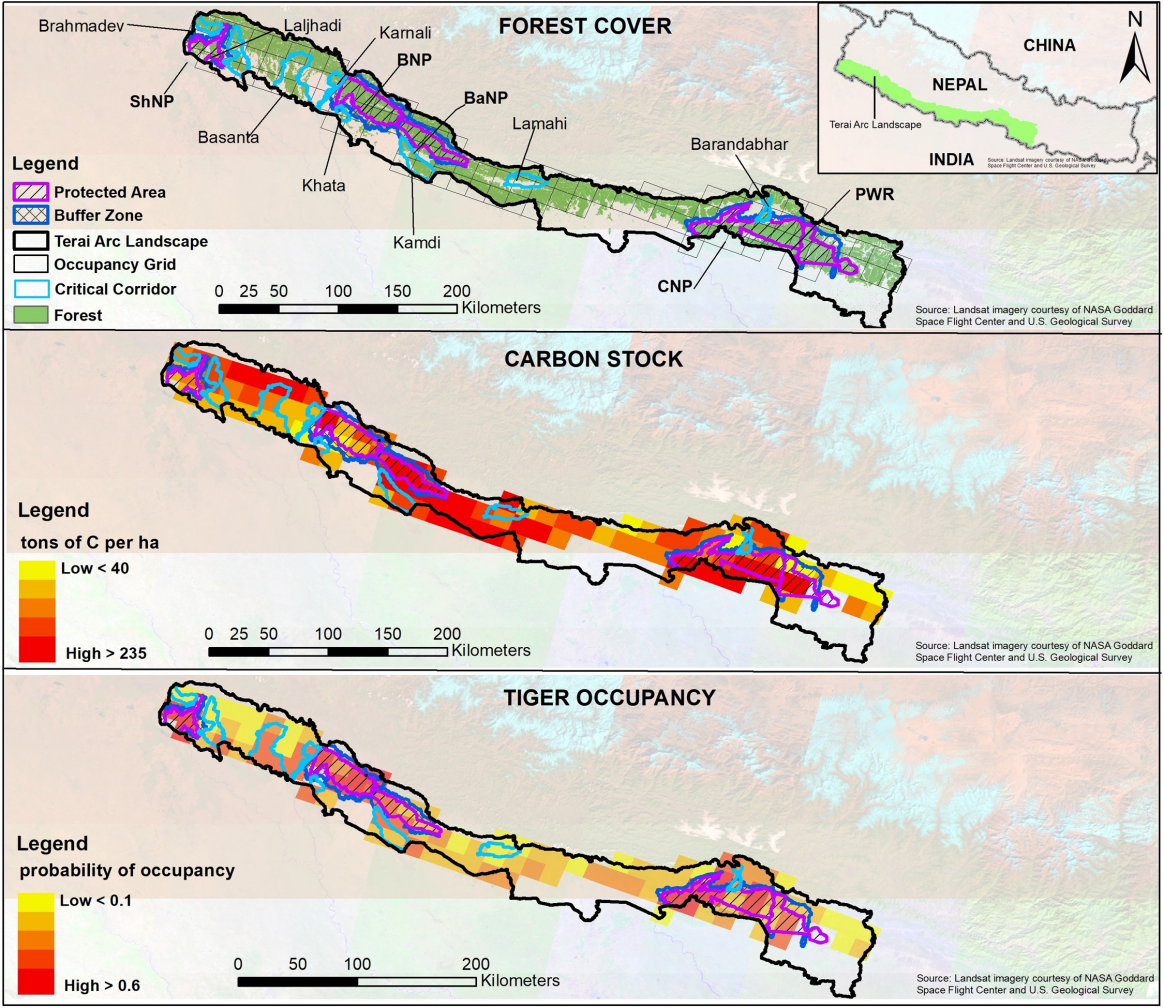
Terai Arc Landscape showing five tiger bearing protected areas along with identified corridors and forest connectivity (in green) (top), above-ground biomass carbon stock (in tons of C per ha) (middle), and tiger occupancy (probability of occupancy, *Psi*) (bottom).

For assessing relationship between AGBCS and tiger density at the protected area level, one of the five tiger bearing protected areas was selected from TAL—CNP (27.16 N—83.50 E to 27.42 N– 84.46 E, 953 km^2^, the oldest national park of Nepal, Fig 1). CNP was selected firstly due to availability of data on AGBCS and posterior tiger density estimates [25]; and secondly, least variation in tiger density estimates in CNP among other tiger bearing protected areas in the landscape. CNP is in the south-central region of Nepal and in the eastern section of the TAL. The national park has a monsoonal humid climate with more than 85% of the annual precipitation (2,180 mm) occurring between July and October. The dry season occurs for 8 months between November and June. The vegetation can best be described as a subtropical, dry, and deciduous forest with colonizing *Saccharum spontaneum* and *Imperata cylindrica* on the dry riverbeds and the floodplains to a climax Sal (*Shorea robusta*) forest on bhabar and hillsides [26]. The national park supports a diverse mammalian fauna including carnivores such as tiger, leopard (*Panthera pardus fusca*), and dhole (*Cuon alpinus*). Tiger density (no. of individuals per 100 km^2^) in CNP is estimated at 3.84 (SD 0.34) in 2013, 3.81 (SD 0.25) in 2018, and 4.06 (SD 0.22) in 2022 respectively, while tiger density in rest of the four tiger bearing protected areas (Parsa, Banke, Bardia and Shuklaphanta National Parks) ranged between 0.97–7.15 tigers per 100 km^2^ [18] in 2022.

To address our objectives, Nepal side of TAL (28.45 N—80.04 E to 27.03 N– 85.25 E, Fig 1) was focused that stretches across 700 km along the flat plain between the Bagmati River in the east to the Shuklaphanta National Park in the west (Fig 1). Entire TAL is extended till Yamuna River in India covering total area of 50,911 km^2^ (Nepal: 24,710 km^2^, India: 26,201 km^2^). Five protected areas (Parsa, Chitwan, Banke, Bardia, and Shuklaphanta National Parks) are located within the Nepal side of TAL with varying degrees of forest connectedness identified as corridors (seven in total: Shikaribas, Barandabhar, Kamdi, Khata, Karnali, Mohana-Laljhadi, and Bramhadev). Corridors were identified based on ground surveys and tiger dispersal model (developed through GIS-based least cost pathway model) developed from last 30 years of field research on tigers [17]. These seven corridors were priority for ecological restoration. Krishnasar Conservation Area is also located in TAL with a focus on Blackbuck conservation and connected to Bardia National Park through Babai River and riverine forests patches in between. Entire TAL is part of the Terai Duar and Savannah Grasslands ecoregion [27] and contains the remaining 75% of forests of the Tarai plains and the Siwalik hills.

### Supervised habitat classification in Terai Arc Landscape

We used remotely sensed supervised classification data [28] for mapping AGBCS at the landscape level as they have been widely used in studies to link AGBCS measurements from the field to satellite observations [29]. We used freely available Landsat 7 Enhanced Thematic Mapper Plus (ETM+) cloud-free mosaic image from the United States Geological Services (USGS)’s Global Land Cover Facility (GLCF) for the year 2001 image having Row Path 141–41, 142–41, 143–41, and 144–40. Shrestha [28] used 75 ground-truthing points for the habitat classification and classified images into 8 habitat classes for classifying all land cover designations and includes: dry sal forest, high density mixed forest, high density sal forest, low density mixed forest, low density sal forest, degraded scrubland, grassland, and riverine forest (See S1 Fig). Sampling frequency of ground truthing points along each habitat class were dry sal forest (19%), high density mixed forest (13%), high density sal forest (12%), low density mixed forest (12%), low density sal forest (12%), degraded scrubland (8%), grassland (11%), and riverine forest (13%). Details on supervised classification analysis can be found elsewhere [17, 28]. Habitat classification was conducted in ERDAS Imagine (Ver.16, Hexagon Geospatial, Atlanta, Georgia) for image processing and classification, while post-classification and a change analysis was done in ArcGIS (Ver 10.2). From the same Landsat 7 ETM+ data sets for our habitat classification analysis, confusion matrix was prepared where overall producer’s classification accuracy to be ~ 82.2% and kappa coefficient to be ~ 0.80.

### AGBCS estimation along the habitat classes

We used the simplest approach [29, 30] to derive AGBCS maps by assigning a single value to each of the habitat classes that have been derived from satellite data (Landsat 7 ETM+) and placed into eight habitat categories. AGBCS was mapped by assigning carbon values for above-ground biomass to each habitat classes (identified as *i*). This is similar approach done for carbon mapping in Sumatra tiger habitat in Indonesia [13]. The carbon stock for each habitat class is equal to the above-ground densities for habitat class as represented in Fig 1. The total carbon storage is equal to the sum of carbon density for each habitat class multiplied by the area for respective habitat class *i* with *n* as the number of habitat classes (identified in middle figure in Fig 1). Carbon stocks estimation in TAL was based on the forest inventory method [20]. Published AGBCS datasets were taken from 97 sampling plot (circular plot measuring 500m^2^) spread across the landscape [20, 31]. Of the total 97 AGBCS sampling plots (*aka* sample size), 53 plots were located across the five national parks (Parsa National Park (n = 7), CNP (n = 20), Banke National Park (n = 14), Bardia National Park (n = 8), and Shuklaphanta National Park (n = 4)), respectively, while rest of the 44 plots were located in identified corridors and large forest connectivity blocks outside the protected areas. Spatial distribution of sampling points along the habitat classes includes degraded scrub (n = 9), dry sal forest (n = 29), high density mixed forest (n = 7), high density sal forest (n = 17), low density mixed forest (n = 15), low density sal forest (n = 14), grassland (n = 5), and riverine forest (n = 1). Only one sampling plot was located in the riverine forest habitat, so published carbon stock representing riverine forest habitat was used-estimated at 80.47 tC/ha [32]-for the analysis.

### Comparing tiger density and AGBCS

We used 2013 published results from camera trap survey conducted in CNP. Camera trap tiger data were collected from 365 camera trap grids-each grid cell measuring 2 km by 2 km-spread across 1,460 km^2^ of tiger habitat (grassland and forest) in CNP. Survey was conducted in winter season (Feb-May) in 2013 with total sampling effort of 10,860 camera trap days [33]. At each grid cell, a pair of cameras were deployed at a strategic locations such as animal trails, forest road, river banks, and human trails etc. for total of 15 days. All the photos from the survey were collected and individual tigers were identified based on strip patterns. Detection history of the individual tigers were made. SECR models [34, 35] were run for the tiger population density estimation. Survey estimated population size of 120 tigers in CNP [33]. For comparing the relationship between tiger density and AGBCS, CNP plot-wise (0.33 km^2^) converged posterior tiger density estimates (no of tigers per 0.33 km^2^) [33] was used. Tiger density estimation in Dhakal *et*. *al*. [33] was carried out using the Bayesian-Spatially Explicit Capture-Recapture (B-SCR) approach [36] implemented in SPACECAP [37]. A grid of home range centers with equally spaced points (with habitat activity centers ~ 9,240 points), each 0.33 km^2^ measuring 4,374 km^2^ was used for tiger density estimation in SPACECAP. The habitat extent of 10 km roughly representing Chitwan valley and part of the Valmiki Tiger Reserve were used, which are historically known to have tiger habitat [38]. After removing the 2,330 km^2^ area of settlements (villages and agriculture areas) and other areas with no carbon data (such as river banks, water bodies, and clouded portion), habitat mask was finalized at 2,044 km^2^ (~6,858 activity centers) for further analysis. Three standard input data files (animal capture dates and locations, trap deployment dates and locations, and hypothetical activity centers) were used. Dhakal *et*. *al*. [33] analysis used the half normal detection function and included a behavioral response in the detection process and performed 65,000 iterations, of which the initial 15,000 were discarded as the burn-in, thinning rate was set at 1, and augmentation value of 350 individuals (over three times the expected number of ~120 animals) for CNP. Geweke diagnostic statistics with |Z| < 1.6 score was used to check model convergence [37]. Using the B-SCR, overall tiger density (no of tiger per 100 km^2^) in CNP estimated to be 3.84 (SE 0.34) with estimate sigma (σ) value equals to 4.1 km. This sigma value infers to tiger activity centers concentrated within 4.1 km from its captured location. Converged posterior tiger density estimates for each habitat pixel taken from the output file of the SPACECAP analysis was used for developing the spatial tiger density surface in ArcGIS 10.2. The spatial location of each habitat pixel, containing posterior tiger density estimates, was overlaid with layer of habitat classes. Carbon stock was computed as the summation of carbon stock of all habitat classes available divided by the total area of habitat classes combined in each pixel.

### Comparing occupancy probabilities and AGBCS

We used tiger occupancy probabilities [23] estimates and compared with total AGBCS for assessing the relationship between metrics at the landscape level. Occupancy survey was carried out across the potential tiger habitat (forest and grassland, measuring 13,915 km^2^) spatially divided into 96 grid cells (each grid cell measuring 15 km by 15 km (~225 km^2^)). We surveyed high probability sites (e.g. trails, ridgelines, roads, and river and stream beds) for tiger signs (scrape, scratch, scats, pugmark, kills, and urine) detection within each grid cell [39].

The survey was completed during the cool-dry period from December 2008 to February 2009 to ensure seasonal consistency across sites with sampling effort of 2016.5 km of walk along the search path. Tiger occupancy data was analyzed in the program PRESENCE [40] for estimating site specific tiger occupancy and detection probabilities. Published site-specific tiger occupancy probabilities [41] (range: ψ _min_ = 0.01 to ψ _max_ = 1.00; ψ_mean_ = 0.366) estimated for 96 grid cells in the landscape was used for the analysis. AGBCS was assigned to each habitat classes available within each grid cell. Carbon stock was computed as the summation of carbon stock of all habitat classes available divided by the total area of habitat classes combined in each grid cell.

### AGBCS in conservation units

Gurung et al. [20] did not segregate the carbon stock based on conservation unit wise- protected areas, corridors, and large forest connectivity blocks. We segregated habitat classes and computed total AGBCS at protected area, corridors, and large forest connectivity blocks level respectively. Spatial layers of five-tiger bearing protected areas, corridors, large forest connectivity blocks, and landscape boundaries were overlaid onto the layer of spatial distribution map containing eight habitat classes datasets (see S1 Fig) in ArcGIS (Ver10.2). The total carbon stock for each conservation unit is equal to sum of its carbon density multiplied by the total area of the conservation unit.

Pearson correlation [42] was used for measuring the relationship between mean tiger density and total AGBCS, while between grid-specific tiger occupancy probabilities and total AGBCS. Mean, cross tabulation, and correlation tests were conducted using IBM SPSS (Ver 22.0) for Windows.

## Results

### AGBCS at sampling plot

**Fig 2:**
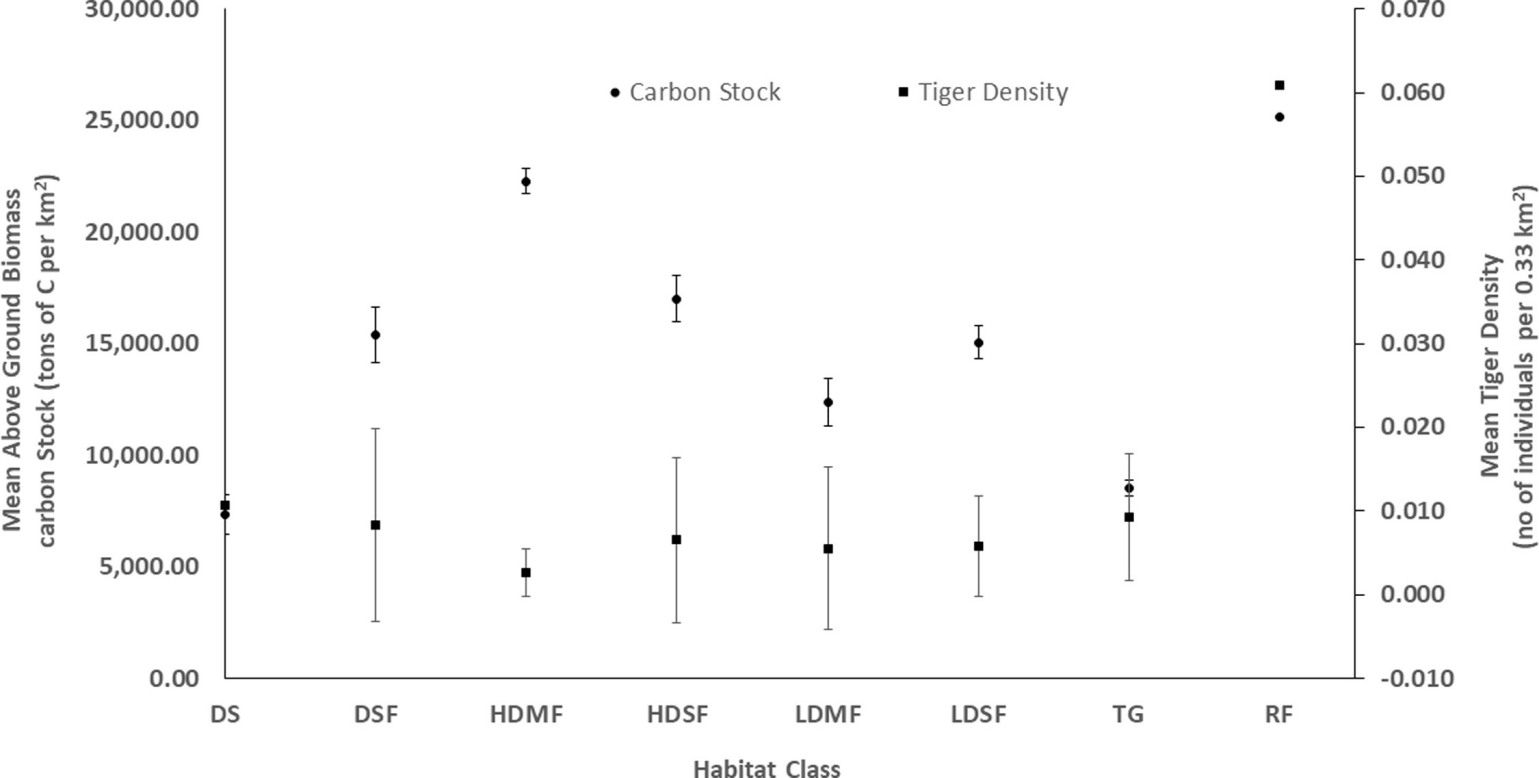
Variation in mean tiger density (no of individuals per 0.33 km^2^) and above-ground biomass carbon stock (tons of carbon per km^2^) along with standard error bars categorized by eight habitat categories. DS: degraded scrub; DSF: dry sal forest; HDMF: high density mixed forest; HDSF: high density sal forest; LDMF: low density mixed forest; LDSF: low density sal forest; TG: tall grassland; RF: riverine forest.

**Fig 3:**
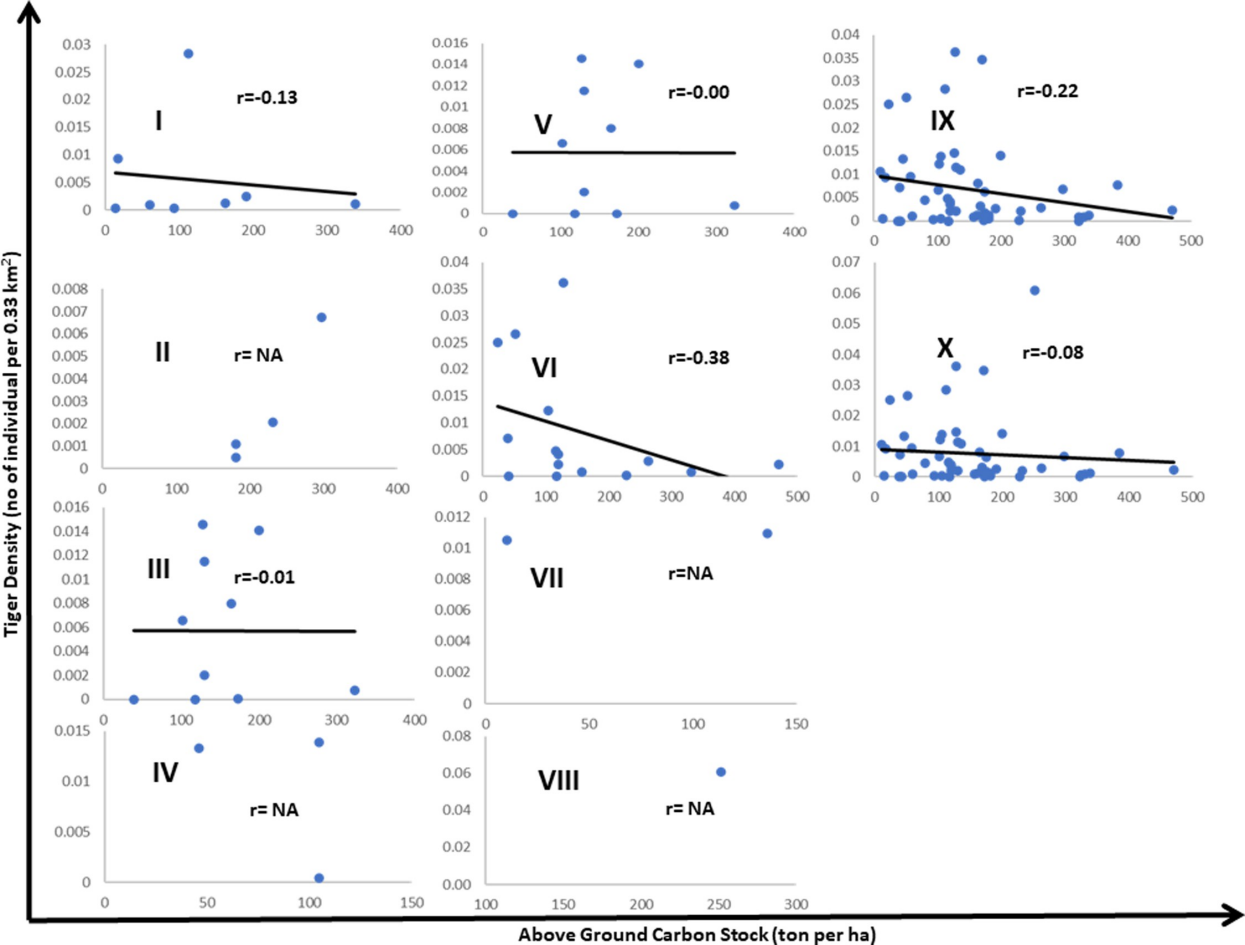
Relationship between above-ground carbon stock and tiger posterior density estimates [33] along four individual forest categories, non-riverine forest, all forest habitat categories in sampling plot (n = 54) within the five protected areas in Terai Arc Landscape. The dotted line represents the trend line. "r” represents Pearson correlation coefficient. NA represent correlation could not ascertain due to low sample size. I: low density mixed forest; II: high density mixed forest; III: high density sal forest; IV: tall grassland; V: low density sal forest; VI: dry sal forest; VII: degraded scrub; VIII: riverine Forest; IX: non-riverine forest habitat; X: overall forest habitat.

We found variation in AGBCS along the mosaic habitat sampling plots (Fig 2) spread across 8-habitat class with high AGBCS recorded in high density mixed forest (~223 tC/ha) and low in degraded scrubland (~73.2 tC/ha). Among the sampling plot in national parks (n = 53), tiger density ranged between 0.003 tigers per km^2^ in degraded scrubland and 0.06 tigers per km^2^ in the riverine forest (Fig 2). We found high variation in negative correlation (r = -0.00 to -0.38) between the total AGBCS and tiger density across the sampling plot (Fig 3).

### AGBCS within Chitwan National Park

**Fig 4:**
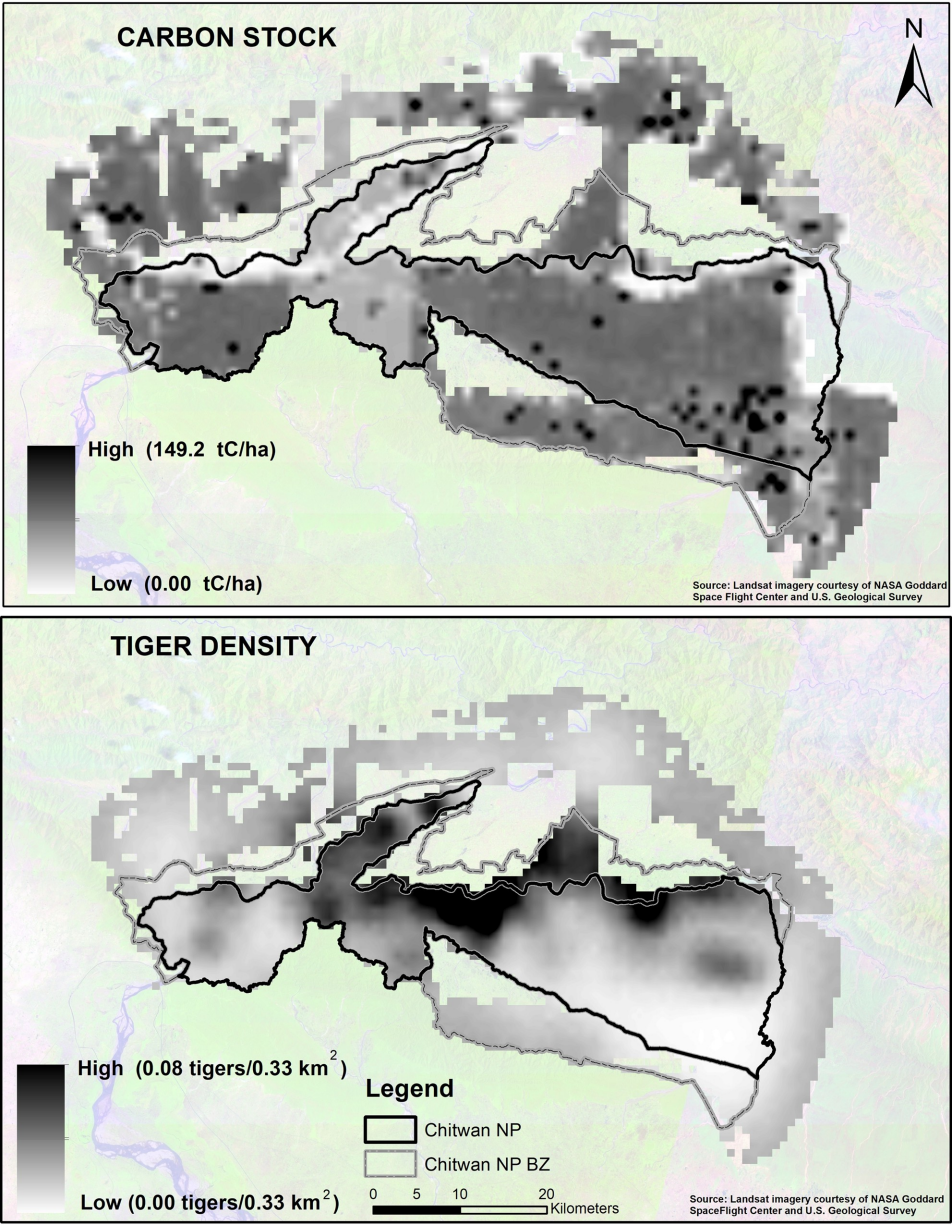
Pixelated density map showing above-ground biomass carbon stock distribution (top) and tiger density (bottom) in Chitwan National Park (NP) and its buffer zone (BZ). Each pixel size is 0.33 km^2^.

In CNP, AGBCS ranged between ~297 tC/ha in high-density sal forest and~46 tC/ha in tall grassland with an average of ~160 tC/ha. All the forests (~1,930 km^2^) and grasslands (114 km^2^) which are potential tiger habitats contains ~ 30.19 million tC. We found a significant negative correlation (n = 6,858, r = -0.20, *p* < 0.01) between tiger density and AGBCS in CNP and surrounding areas. We found highest tiger density ~ 0.06 individuals per 0.33 km^2^ in the riverine forest and lowest estimates (~0.00) in degraded scrubland, respectively. At the fine pixel level, overall mean tiger density of 0.0135 tigers per 0.33 km^2^ is equivalent to mean total of 43.7 tC/ha (range: 149.2–0.003 tC/ha) in CNP (Fig 4).

### AGBCS in the protected areas, corridors, large forest connectivity, and landscape

**Fig 5:**
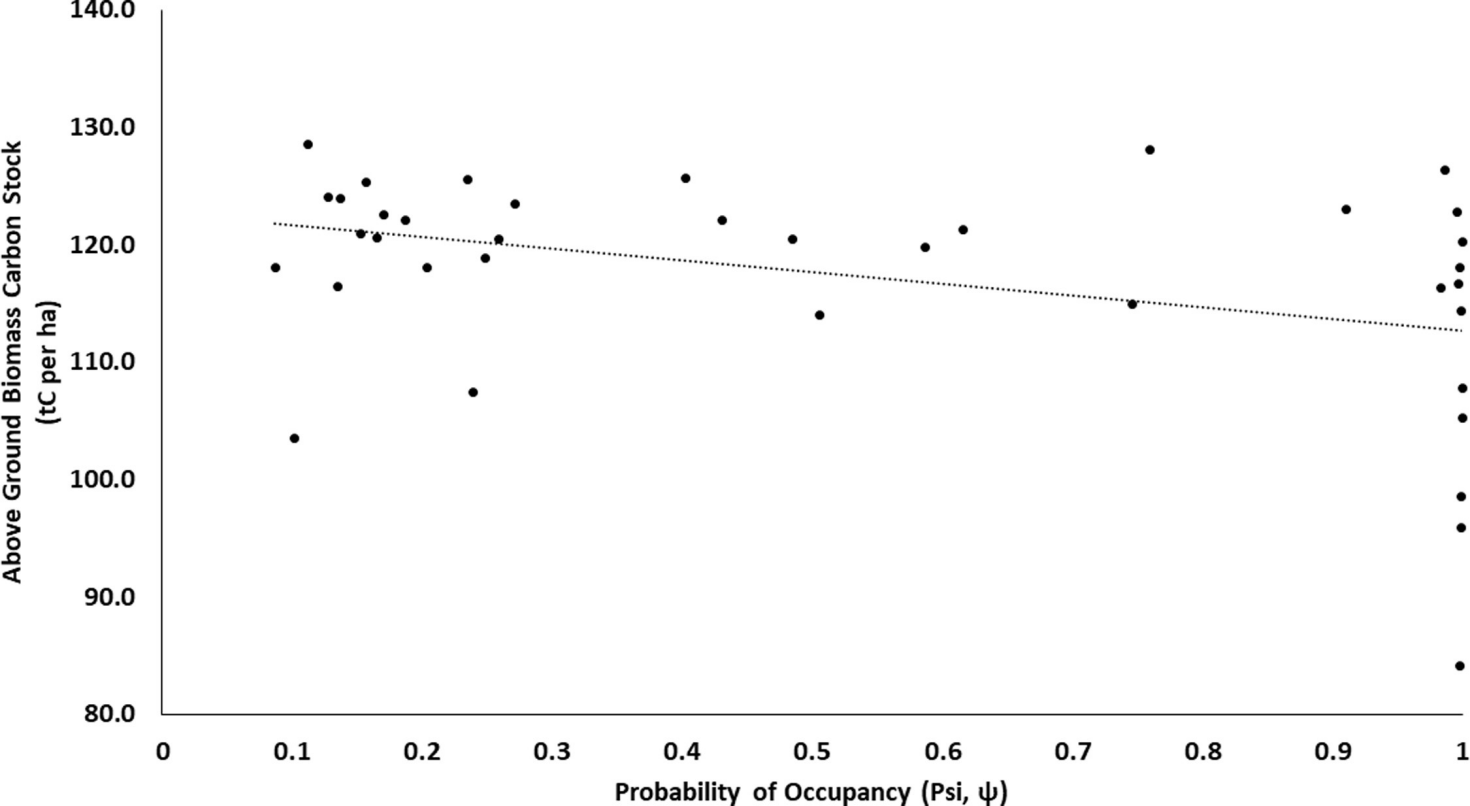
Relationship between above-ground biomass carbon stock and the probability of tiger occupancy (after Barber et al. [41]). The dotted line represents the trend line.

Grid wise AGBCS ranged between a minimum of 72.9 tC/ha (within the corridors) to the maximum of 128.7 tC/ha (within protected areas) at the landscape level (Fig 1). We found a significant negative relationship (r = -0.24; *p* = 0.023) between grid cell-specific tiger occupancy probability and AGBCS at the landscape level (Fig 5). In a total of 96 grid cells containing 11,087 km^2^ of potential habitat, total AGBCS estimated at 130.4 million tC with a mean occupancy probability estimate of 0.366 (SE 0.02).

Tiger bearing protected areas, corridors, and large forest connectivity blocks contain 56.7 million tC (~119 tC/ha), 33.0 million tC (~117 tC/ha), and 40.6 million tC (~117 tC/ha), respectively. The probability of tiger occupancy (*Psi*, ψ) ranged between: (1) ψ_min_ = 0.08 and ψ_max_ = 1.00 for protected areas; (2) ψ_min_ = 0.04 and ψ_max_ = 0.63 for corridors; and (3) ψ_min_ = 0.01 and ψ_max_ = 0.33 for large forest connectivity blocks, respectively.

## Discussion

This is the first study to document and explore the relationship between the estimates of AGBCS and tiger population metric (population density & occupancy probability). The major findings of the study have been 1) significant negative relationship between tiger density & AGBCS in CNP, and between tiger occupancy & AGBCS in the landscape level, 2) tiger density of 0.0135 tiger per 0.33 km^2^ is equivalent to mean total of 43.7 tC/ha in CNP, 3) mean landscape occupancy ψ = 0.366 is equivalent to mean AGBCS of 116 tC/ha in the landscape, and 4) Comparatively, site-specific variation in tiger occupancy and its equivalent AGBCS: medium in tiger corridors and large connectivity dispersal blocks, while high in tiger bearing protected areas.

Although the data set was part of the Gurung et al. [20], analysis was conducted at different scales (plot, protected areas, and landscape) makes a variation in AGBCS findings but comparable result between the studies. As expected, our AGBCS estimated for protected areas (including CNP) ranged between 150–156 tC/ha falls within lower range of estimates 178 ± 42.63 tC/ha (Gurung *et*.*al*. 2015). Similarly, the mean AGB reported here in different conservation unit, largely within subtropical climate, can be comparable as highlighted in Bordoloi *et*. *al*. [43] with the AGB estimates of 16.76 to 173.72 Mg/ha (18.47–191.5 tC/ha) from subtropical forest of Chenzhou City in China [44] and 160 Mg/ha (176.37 tC/ha) reported from subtropical community forest of Nepal [45]. Variation in AGBCS, yet comparable, along the potential tiger habitat differed between conservation units. AGBCS with 119 tC/ha in protected areas falls under the strict protection regime, while large forest connectivity blocks and corridor at 116 tC/ha falls under the combination of protection and utilitarian regime. We found more average AGB carbon (~3 tC/ha) in protected areas, albeit by low margin, than in non-protected areas regime. Among various reasons including composition and extent of patches, their protection measure status for protecting carbon biomass could be one factor [46] for more AGBCS within protected areas. Non-protected area regimes (corridors and large forest connectivity blocks) allow the harvesting of the forest such as fuelwood, timber, and non-timber forest products for the discrepancy in carbon content. This also supports the argument that carbon stock is higher in protected areas than in non-protected areas highlighted in many studies in Southeast Asia [47], India [48], and Nepal [49]. The Government of Nepal’s target to increase the protection regime to 30% by 2030 from the current 24% and maintaining 45% of total area under forest cover as committed in country position paper under Global Biodiversity Framework in CoP 15 in 2022 and Second Nationally Determined Contributions submitted to the International Paris Agreement in CoP 24 in 2020. These commitments and targets are positive steps in increasing carbon content in its policy targets and shall contribute both to, biodiversity conservation and carbon sequestration, respectively.

There was a negative relationship between tiger density & occupancy probability with AGBCS. Our observation on the inferred relationship between AGBCS and tiger density & occupancy probability seems reasonable and obvious. Tiger population parameters (such as density and occupancy estimates) are dependent upon prey density [39, 50]. Ungulate density is high in the riverine complex and generally low in the sal forest [51] across the TAL-Nepal. The clear positive relationship between tiger density and prey density (Karanth et al. 2004) supports this contention. For any carnivores including tigers, habitat selection is a key process shaping ecological communities through predator-prey interactions [52]. It is to be noted that with the exclusion of the riverine subset, albeit weak negative relation persists, more transition towards the positivity in relationship (r = -0.22 to -0.08) between AGBCS and tiger density. Thus, the contribution of riverine forest (~2% coverage) in the landscape is subtle yet extremely important for the survival of the tiger and its prey and other species of global importance megafauna such as greater one-horned rhinoceros (*Rhinoceros unicornis*). *Trewia nudiflora*-*Bombax ceiba-Acacia spp*. assemblages in riverine forest and mixed forest types are identified along riparian zones in association with riverine grasslands assemblages [53].

We found site-specific variations: high carbon /low-population density & occupancy sites, low carbon/high-population density & occupancy sites along with the various habitat types including grassland collectively identified as potential tiger habitat. Carbon conservation needs to keep harmony in managing between pioneer forest (dry) to riparian zones (wet) for maintaining healthy tiger density and occupancy in the landscape. Protected area is managed as per the management plan where enriching biodiversity conservation is a priority. Active habitat management in low carbon-rich ecosystems such as floodplain grassland including rich riverine forest and degraded scrubland can be managed to boost prey population for enriching tiger populations [54, 55]. In corridors, carbon-rich forests can be managed as per local needs co-benefiting local diversity by taking an integrated approach [56, 57]. Khata corridor in Western TAL takes in an integrated approach with sustainable management of carbon-rich forest and benefiting the local biodiversity that also facilitates the tiger dispersal between protected areas [25]. As a caveat, the study explored the relationship between the metrics the tiger population and AGBCS and, results alone should not be treated as management treatments for enriching tiger recovery or carbon stock per se.

Tiger densities are high along the riverine and floodplain grassland habitat [51], thus maintaining the riverine and grassland habitat does support conservation of AGBCS, albeit low in content, with mutual benefits to tiger conservation. River valley corridors along the plains and mid-hills do provide an opportunity for enhancing AGBCS and conserving the habitat as a refuge for dispersing tigers. Riverine forest and grasslands along river valleys in East Rapti, Narayani, West Rapti, Babai, Karnali, and Mahakali Rivers are linked to sources sites of tigers such as Chitwan, Banke, Bardia, and Shuklaphanta National Parks and are potential to increase a AGBCS while providing habitat for their dispersal specially under climate change when most other areas could be less inhabitable.

## Conclusions

This study assessed forest carbon status along the conservation units defined for tiger conservation such as corridors, large forest connectivity blocks, and protected areas and explored relationship carbon and tiger conservation in terms of above-ground biomass carbon stock, tiger population density, and occupancy probabilities. It has been observed that maintenance/restoration of existing forest habitat and/or mosaic habitat (riverine forest and grassland) would still be preferable for both tiger (0.06 per 0.33 km^2^) and carbon conservation (80.47 tC/ha). Within the CNP, inducting the activity centers of tigers (sigma value ~4.1 km) provide extent of the all the tiger captured with the national park covering range of habitat (~2,044 km^2^) and protection of these potential habitat provides opportunity to conserve rich carbon (~ 30.19 mtC). Management of grassland habitat is critical for recovery of tiger prey population. Result shows that grassland stores relatively less biomass carbon than other habitat type. However, grassland habitat also stores highest amount of soil carbon relative to other habitat types such as forest [58, 59]. Considering carbon stock from biomass and soil, CNP grassland habitat represent more resilient carbon sinks and could store more carbon, comparable to other habitat types. Further empirical research is anticipated considering carbon stock above and below the surface in this space.

Government of Nepal’s periodic species conservation action plan for major flagship species including tiger can also integrate carbon conservation potential while managing habitat through strategic actions. This urged to species- and site-specific management strategy that also focuses on habitat management plans on riparian habitat (forest and grassland) and low transitional state forest (degraded scrubland) providing opportunities to generate win-win solution, sequester more carbon, and provide better habitat for tiger.

We note that our study design has drawn a relationship from limited sampling data, covering limited area and habitat types, but we used a published dataset for elucidating total carbon stocks. More research using larger sample size, covering larger area and habitat types, is required to determine if our findings are indicative of broader area and elucidating biological reason for cause-and-effect relationship.

## Supporting information

S1 Fig Spatial distribution of 8 identified habitat types (after Shrestha [28]) in Terai Arc Landscape-Nepal. Map segregated into three parts: Upper Western complex of TAL- Middle Central complex of TAL- Lower Eastern complex of TAL.
(JPG)
