# Supplementary figures and images for: Carbonated tiger-high above-ground biomass carbon stock in protected areas and corridors and its observed negative relationship with tiger population density and occupancy in the Terai Arc Landscape, Nepal
Source: PLoS One. 2023 Jan 25;18(1):e0280824. doi: 10.1371/journal.pone.0280824 (PMC9876270; doi:10.1371/journal.pone.0280824)

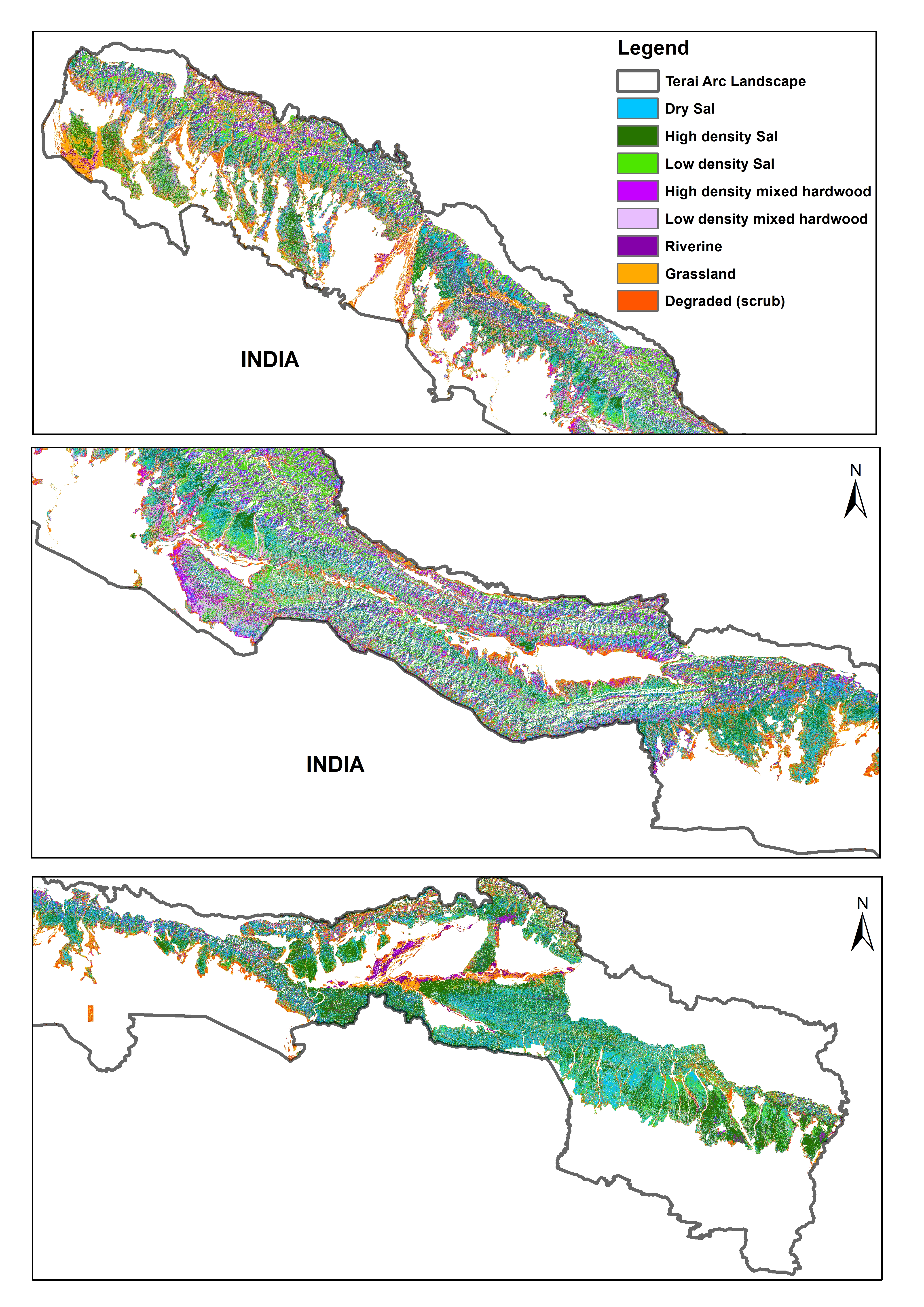

Supplement: S1 Fig — Map segregated into three parts: Upper Western complex of TAL- Middle Central complex of TAL- Lower Eastern complex of TAL. (JPG) [file pone.0280824.s001.jpg]
